# A Dictionary of Terms Used in Medicine and the Collateral Sciences

**Published:** 1855-11

**Authors:** 


ART. XI.—*A Dictionary of Terms used in Medicine and the Collateral Sciences.* By RICHARD D. HOBLYN, A. M., Oxon. A new American from the last London edition. Revised, with numerous additions, by ISAAC HAYS, M. D., Editor of the American Journal of the Medical Sciences. Philadelphia: Blanchard & Lea. 1855.

The most unpretending of lexicons—a neat duodecimo volume of 520 pages, closely printed and presenting great exactness, with brevity in the synonyms and definitions. The editor's preface so well describes the work, that we avail ourselves of its language:

"Believing that its republication in this country would be useful, the Editor consented to revise and adapt it to the wants of the American practitioner. With this view he has added, not only the terms recently introduced, but also the names of our native medicinal plants,—the formulæ for the officinal preparations, &c.,—and has made the work conform with the latest edition of the Pharmacopœia of the United States. For the greater convenience of reference, he has also inserted in the body of the work most of the interesting articles placed by the author in an *Appendix;* and also the Terms contained in the "*Supplementary List*" to the last London edition, with the exception of those under the first few letters of the alphabet, which have been appended in a separate list. To accommodate these additions, not only has the size of the page been materially enlarged, but also the number of pages has been increased by more than one hundred.

"The aim of the Editor has been to render the work more complete, not by incorporating in it obsolete words, but by adding such as modern investigations and doctrines have introduced, so that the student should be afforded an explanation of all the terms at present in use."

